# Cinchona Alkaloids and Their Salts

**Published:** 1877-06

**Authors:** Ferdinand King

**Affiliations:** Atlanta, Ga.


### CINCHONA ALKALOIDS AND THEIR SALTS.

By FERDINAND KING, M.D., Ph.G., Atlanta, Ga.

Owing to the scarcity and high price of quinine, physicians have for some time past been experimenting with quite a number of alkaloids of various kinds, in the hope of finding a good substitute for that time honored and well known remedial agent. These experiments and investigations which have been more vigorously prosecuted in the western and southern States than elsewhere, have not been confined exclusively to the small group of alkaloids derived from cinchona, but have embraced every other remedy calculated to produce an anti-periodic or febrifuge effect upon the animal economy. In searching for this much coveted substitute our profession has been sadly imposed upon by large numbers of silver-tongued agents sent out by unscrupulous, enterprising manufacturers who by their smiles and graces induce some leading physicians to give their preparations a trial, thus creating a temporary demand for them, thereby enriching the originators and making consumers poorer and very little wiser. In my humble judgment we lose ground every time we deviate from the old and long beaten track. The scientific investigator so far has been unable to find anything possessing the same anti-periodic properties as cinchona and its preparations. Some new discoveries have for a time enjoyed considerable reputation, but after thorough tests, have gone down before cinchona and its allies, never to rise again.

Sometimes we have unpleasant symptoms, such as urticaria, following the administration of quinine, which I am sure depends upon some idiosyncrasy of the patient, just as catarrhal symptoms not unfrequently result from the use of iodide of potassium. These consequences observable in such a small per cent. of patients where valuable therapeutic agents are administered, should not, I am sure, prejudice us against their general use. The same objections urged against the use of quinine under certain pothological conditions can with equal propriety be applied to the other alkaloids of cinchona-quinidia, cinchonidia, quinidia and cinchonia. These unpleasant effects are observable when we administer any of the alkaloids just enumerated, in large doses. Take for example, sulphate of cinchonida, which is perfectly worthless as an anti-periodic unless we give at least three times as much of it at a dose, as we do of the quinine. When given in these large doses we find all the symptoms of true "quinenism" present—such as cerebral disturbance evinced by a feeling of tightness in the head, ringing in the ears, difficulty of hearing, etc. It seems that these effects must be secured before the system of our patient can be considered proof against repeated malarial paroxysms. As it requires three or four times as much sulphate of cinchonidia for example, to secure this "quininism" as it does of the sulphate of quinine, I think we practice poor economy in administering the former in preference to the latter.

I do not think the *modus operandi* by which the various alkaloids of cinchona relieve malarial fevers or influences is really understood by a large majority of our profession, but that it is too often hypothetical in the minds of our average practitioner. That some, and perhaps many, of these hypotheses are correct, I do not pretend to deny. We all know that the blood undergoes the most remarkable changes in patients suffering from the effects of malarial poison. There is a very perceptible increase in the quantity of the plasma or alkaline fluid, while there is a corresponding decrease in the red globules or corpuscles. This condition is plainly evinced by the characteristic paleness always observable in patients suffering from malarial anæmia. Scientific and intelligent physicians will readily agree that it is necessary to restore the blood to its normal condition before we can give back to our patient his wonted health. We should therefore examine his blood, and learn what elements are wanting, before we attempt to administer remedies. We should then give him such medicinal agents as are calculated to replace the lost elements and restore the blood to its original healthy condition. We find nothing in our materia medica that meets all the indications in these cases as completely as the cinchona or Peruvian bark itself, but owing to its bulk and the length of time required for its assimilation, it is objectionable. The ingenunity of the intelligent pharmacist has overcome these objections by giving us all the active principles of the Peruvian bark in a combination very appropriately called by its manufacturers (Messrs.

Billings, Clapp & Co., of Boston) *cincho-quinine*. In this combination we find the nearest approach to the original substance, Peruvian bark, that modern science has yet attained. It is, as before stated, a combination of all the active medicinal principles of the best calisaya bark; and, after the long and thorough testing it has had, it stands unrivalled as a prompt, safe, and uniformly reliable antiperiodic, possessing all the advantages, with none of the disadvantages, of sulphate of quinine, or any of the single alkaloids of the cinchona. It is entirely free from such external agents as sugar, liquorice, starch, magnesia, etc. It is wholly composed of the bark alkaloids, quinia, cinchonia, quinidia, cinchonidia, and the other alkaloidal principles which have not been distinctly isolated, and the precise nature of which is not well understood. Analyses by disinterested chemists attest their presence. In its preparation all the active tonic and febrifuge principles of the bark are secured without the inert bulky lignin, gum, tannic acid, etc. It exerts the full therapeutic influence of sulphate of quinia, in the *same dose*, without oppressing the stomach or creating nausea. It seldom produces cerebral distress, as quinine does, and I have found it to produce much less constitutional disturbance than the latter agent.

During my experience as a country doctor, in the swamps of Alabama, I found it far superior to any other preparation I ever used in the treatment of the chronic forms of chills found there. I could not cure my patients with the quinine uncombined, as it simply acted as a cerebral stimulant, and didn't seem to improve the debilitated condition of the sufferer, as did cincho-quinine. I have never found any difficulty in getting the most delicate child or squeamish female to swallow the cincho, as it is almost tasteless and does not leave that clinging, lasting, bitter taste peculiar to the sulphate.

One of the main points I desire to impress upon the minds of those who read this article is, that cincho-quinine is not sulphate of quinine; it is not sulphate of cinchonidia, but it is all the alkaloids in *combination*, and it is this composition, this representation of all the medicinal principles found in Peruvian bark that gives it the value claimed for it over and above all other preparations, or any one of the alkaloids.

It is generally believed by a large number of old practitioners that the reason why larger quantities of quinine are required to combat malarial diseases than formally, is not because of the impurity of the sulphate of quinine but because it is too purely and solely a sulphate lacking the other useful and important principles above referred to, and that are so surely calculated to successfully overcome the various unfavorable pathological conditions that invariably present themselves in patients suffering from exposure to malarial or miasmatic influences.

Cincho-quinine is not explosive in its action, as in the *combination* the amount of nitrogen always present in most alkaloids, is greatly diminished by uniting the several cinchonia alkaloids, hence less cerebral feelings and less constitutional disturbance follow its administration than are usually attendant upon the use of the uncombined alkaloids. The same rules as I before stated, are to be observed with regard to the use of the cincho-quinine that govern the use of the sulphate. It is given in the same doses and where the *siege* treatment is indicated, it commends itself to the special attention of the entire medical profession.

Cincho-quinine has been thoroughly tested by many of the most learned teachers and practitioners of medicine in the north and west, and in some sections of the southern States, giving in every instance the most satisfactory results. Indeed I have heard of it being abandoned in no instance, while sulphate of cinchonidia and other cheap alkaloids have gone into total disuse.

Not only should the real value of cincho-quinine recommend the article to our profession, but its low price is another inducement for us to prescribe it. It is less costly than the sulplate while the dose is the same. The price of it fluctuates with the rise and fall of the Peruvian bark just as in the case of the sulphate, still it is at times furnished at about half the cost of the latter article. Some may ask why the cincho-quinine has not come into more general use in the south. To such I would say, it is from the fact that it has been but recently offered to the profession, and has not been so extensively advertised as sulphate cinchonidia and some of the other more worthless articles that have been forced into the offices of a large number of our physicians. Cincho-quinine stands upon its own merits, and will come into more general use at an early day. I know personally, at this comparatively early day, several physicians in our own State, who use it altogether in preference to quinine or any of the single alkaloids. It is not pushed upon our profession by an army of smiling agents that infest our country, singing songs and tipping social glasses with the liberal members of our medical societies at their local or State meetings. Its manufacturers claim no special praise for giving us this valuable and economical remedy. And here I would state—rather parenthetically, that "we are under lasting obligations" to *no living firm* of manufacturing chemists for the cinchona alkolides, as they were all discovered even prior to 1835. The praise for scientific discoveries should, in my humble judgment, be awarded the discoverer, and not him who profits and grows rich by it.

